# MiRNA-146a polymorphism increases the odds of malaria in pregnancy

**DOI:** 10.1186/s12936-019-2643-z

**Published:** 2019-01-14

**Authors:** Welmoed van Loon, Prabhanjan P. Gai, Lutz Hamann, George Bedu-Addo, Frank P. Mockenhaupt

**Affiliations:** 1 Institute of Tropical Medicine and International Health, Charité-University Medicine Berlin, Campus Virchow-Klinikum, Augustenburger Platz 1, 13353 Berlin, Germany; 2 Institute of Microbiology and Infection Immunology, Charité-University Medicine Berlin, Hindenburgdamm 30, 12200 Berlin, Germany; 3 Komfo Anokye Teaching Hospital, School of Medical Sciences, Kwame Nkrumah University of Science and Technology, Kumasi, Ghana

**Keywords:** Malaria, Pregnancy, *Plasmodium falciparum*, MiRNA-146a, Polymorphism, Innate immunity

## Abstract

**Background:**

*Plasmodium falciparum* infection during pregnancy is a major cause of poor maternal health, adverse foetal outcome and infant mortality in sub-Saharan Africa. Genetic disposition is involved in susceptibility to malaria in pregnancy and its manifestation. MicroRNAs (miRNAs) influence gene regulation including that of innate immune responses. A miRNA-146a rs2910164 G > C single nucleotide polymorphism (SNP) has been associated with increased risks of several diseases, but no data as to malaria are available.

**Methods:**

The association between miRNA-146a rs2910164 and *P. falciparum* infection among 509 Ghanaian women attending antenatal care (ANC) and 296 delivering Ghanaian primiparae was investigated. Malaria parasites were diagnosed by microscopy and PCR. Leukocyte-associated hemozoin in placental samples was recorded as well. Proportions were compared between groups by Fisher’s exact test, and logistic regression models were used to adjust for possible confounders.

**Results:**

By PCR, *P. falciparum* infection was detected in 63% and 67% of ANC attendees and delivering primiparae, respectively. In both groups, two in three women were either heterozygous or homozygous for miRNA-146a rs2910164. Among ANC attendees, homozygosity conferred increased odds of infection (adjusted odds ratio (aOR), 2.3; 95% CI, 1.3–4.0), which was pronounced among primigravidae (aOR, 5.8; 95% CI, 1.6–26) but only marginal in multigravidae. Likewise, homozygosity for miRNA-146a rs2910164 in primiparae increased the odds of past or present placental *P. falciparum* infection almost six-fold (aOR, 5.9; 95% CI, 2.1–18).

**Conclusions:**

These results indicate that SNP rs2910164 G > C is associated with increased odds for *P. falciparum* infection in first-time pregnant women who are considered to lack sufficient acquired immune responses against pregnancy-specific strains of *P. falciparum*. These findings suggest that miRNA-146a is involved in protective malarial immunity, and specifically in the innate component.

## Background

*Plasmodium falciparum* infection during pregnancy is a major cause of poor maternal health, miscarriage, stillbirth, low birth weight (LBW), preterm delivery and infant mortality in sub-Saharan Africa. Primiparous woman exhibit an increased susceptibility to *P. falciparum* infection and consequently bear a higher risk for placental malaria (i.e., parasites and/or malaria pigment (hemozoin) discernible in placental tissue or blood), malarial anaemia and malaria-related morbidity and mortality as compared to multigravidae. The increased risk of malaria and complications is largely due to parasites exhibiting specific variants of the *P. falciparum* erythrocyte membrane protein-1, which facilitate adhesion to the syncytiotrophoblast (the surface lining the placental intervillous space), followed by the accumulation of infected erythrocytes and inflammatory cells in the placental intervillous space [1]. The acquisition of specific immune responses to syncytiotrophoblast-adhering *P. falciparum* strains increases with every consecutive pregnancy, resulting in better parasite recognition and reduced susceptibility and manifestation in multigravidae [2]. Moreover, due to placental sequestration, microscopy strongly underestimates actual prevalence of *P. falciparum* infection in pregnancy [3].

Host genetic variation plays an important role in susceptibility to and manifestation of malaria. The association of single nucleotide polymorphisms (SNPs) in genes encoding toll-like receptors (TLRs) and other members of the innate immune system with susceptibility to (severe) malaria in Sub-Saharan African populations [4, 5] suggests that SNPs in other immune regulators such as micro-RNAs (miRNAs) influence malaria as well. MiRNAs are a class of small, non-coding, evolutionarily conserved RNA strains of approximately 22 nucleotides, and they are involved in gene regulation by their posttranslational action at the 3′-UTR region of mRNA. They control many processes, including pathways in the innate and adaptive immune responses [6]. MiRNA-146a is involved in the innate immune response by a negative feedback loop including two key molecules downstream of the TLR machinery: interleukin-1 receptor-associated kinase (IRAK)-1 and TNF receptor-associated factor (TRAF)-6 [7]. Recent studies have shown the potential of using miRNA-146a as a biopharmaceutical agent [8, 9]. The presence of the variant C-allele in SNP rs2910164 disrupts miRNA-146 processing and leads to altered IRAK-1 and TRAP-6 expression [7]. SNP rs2910164 in the passenger strand of pre-miRNA-146a has been linked with both decreased and increased risk to various types of cancer [10], autoimmune diseases [11] and increased susceptibility of mycobacterial infections [12, 13].

In this cross-sectional study, the presence of miRNA-146a SNP rs2910164 G > C was hypothesized to affect susceptibility to *P. falciparum* infection. *Plasmodium falciparum* infection was assessed and the miRNA-146a SNP was genotyped in 805 Ghanaian pregnant women, a group at high risk of malaria.

## Methods

In November and December 1998 and between January 2000 and January 2001, respectively, 530 pregnant women attending antenatal care (ANC) and 893 delivering woman were recruited at the Presbyterian Mission Hospital in Agogo, Ashanti Region, Ghana, a region holoendemic for *P. falciparum* [14]. Informed consent was obtained from all study participants (from parents or guardians of those < 18 years of age). The study protocols were reviewed and approved by the Committee on Human Research Publication and Ethics, School of Medical Sciences, University for Science and Technology, Kumasi, Ghana. Study groups, procedures and malariological indices have been described previously [15, 16]. Briefly, all women were clinically examined, socioeconomic data, gravidity or parity, fever (≥ 37.5 °C, axillary for ANC attendees and sublingual for delivering women) were documented, and samples of venous and intervillous (delivering women) blood were collected into EDTA. For the present study, 530 ANC attendees and 304 primiparae with live singleton delivery were included. The group of ANC attendees comprised of 24.9% (127/509) primigravidae, 21.2% (108/509) secundigravidae and 53.8% (274/509) multigravidae.

*Plasmodium* parasite density in venous and intervillous blood samples were microscopically counted on Giemsa-stained think films *per* 500 white blood cells (WBC) and *per* 100 high-power fields (HPF), respectively. The presence of leukocyte-associated hemozoin in the intervillous samples was also recorded. For ANC attendees, WBCs were counted using a Cell Counter (HC555, Clinicon, Germany) and the peripheral blood parasite density was calculated as parasites per microliter, deducing the multiplicator from the individual WBC count. Plasma and blood cells were separated by centrifugation. Genomic DNA was extracted from blood (QIAamp Blood Kit, Qiagen, Germany) and plasmodial infections and species were diagnosed by nested PCR assays [17]. “Past or present placental malaria” was defined as positivity of placental samples for *P. falciparum* infection by PCR, microscopy, and/or hemozoin detection. MiRNA-146a SNP rs2910164 genotyping was carried out by melting-curve analysis applying commercially available primers and probes (TIB Molbiol, Germany).

Haemoglobin (Hb) was measured by a HemoCue photometer (Ångelholm, Sweden), and anaemia was defined as Hb level < 11 g/dL [18]. Gestational age was assessed within 24 h of delivery by applying the morphological Finnström score and a value < 37 weeks was categorized as preterm delivery [19]; LBW was defined as < 2500 g. Pyrimethamine (PYR), then used as chemoprophylaxis, was detected by enzyme-linked immunosorbent assay based methods in urine (ANC attendees) or plasma (primiparae) [15]. Proportions of *P. falciparum* infection among women with and without the miRNA-146a SNP were compared by a two-tailed Fisher’s exact test, and odds ratio (OR) and 95% confidence intervals (95% CIs) were computed. Additionally, miRNA-146a genotypes were compared with respect to the outcomes of malaria, i.e., fever, anaemia, LBW and preterm delivery. Trends, e.g., increasing infection prevalence in women with wild type alleles over heterozygosity to homozygosity for the miRNA-146a SNP, were tested by the Cochran Armitage test. Logistic regression models were used to adjust ORs of infection for known predictors, i.e., age (years), presence of PYR, and rainy or dry season (only in delivering women). All analysis was done in R version 3.4.3. A *P*-value < 0.05 was considered statistically significant.

## Results

Typing of the miRNA-146a SNP was successful in 96.0% (509/530) of ANC attendees and 97.4% (296/304) of delivering women. The miRNA-146a polymorphism was present in 67.7% (heterozygous, 48.1%; homozygous, 19.6%; allele frequency, 0.44) of ANC attendees and 69.2% (48.3% and 20.9%; allele frequency, 0.45) of delivering women (Tables 1, 2, 3). In both groups, allele distribution was in Hardy–Weinberg equilibrium (c^2^ = 0.24; *P* = 0.62, c^2^ = 0.18; *P* = 0.67).

**Table 1:** Prevalence of *P. falciparum* infection (PCR) according to miRNA-146 genotype in pregnant women attending ANC

SNP rs2910164	Positive cases	Univariate analysis^a^		Multivariate analysis^b^	
	% (Fraction)	OR (95% CI)	*P*	aOR (95% CI)	*P*
All	63.3 (322/509)				
Wild type	55.5 (91/164)	1		1	
Heterozygote	64.9 (159/245)	1.5 (1.0–2.3)	0.063	1.4 (0.9–2.1)	0.12
Homozygote	72.0 (72/100)	2.1 (1.2–3.7)	0.0089	2.3 (1.3–4.0)	0.0053
Het. or Hom.	67.0 (231/345)	1.6 (1.1–2.4)	0.014	1.6 (1.1–2.4)	0.023
Primigravidae	74.0 (94/127)				
Wild type	60.0 (21/35)	1			
Heterozygote	75.4 (46/61)	2.0 (0.8–5.5)	0.17	1.8 (0.7–4.7)	0.20
Homozygote	87.1 (27/31)	4.4 (1.2–21.0)	0.025	5.8 (1.6–26.0)	0.012
Het. or Hom.	79.3 (73/92)	2.5 (1.0–6.4)	0.040	2.5 (1.0–6.2)	0.040
Multigravidae	59.7 (288/382)				
Wild type	54.3 (70/129)	1		1	
Heterozygote	61.4 (113/184)	1.3 (0.8–2.2)	0.24	1.3 (0.8–2.1)	0.29
Homozygote	65.2 (45/69)	1.6 (0.8–3.0)	0.17	1.8 (0.9–3.4)	0.082
Het. or Hom.	62.5 (158/253)	1.4 (0.9–2.2)	0.12	1.4 (0.9–2.2)	0.14

Allele frequencies of SNP rs2910164 G > C were 0.47 (303/644) in infected and 0.38 (142/374) in non-infected women (*P* = 0.005)

*OR* odds ratio, *aOR* adjusted odds ratio

^a^Fisher’s exact test for independence, compared to reference (wild type)

^b^Logistic regression model, including co-predictors age, PYR in urine or plasma and number of antenatal care visits. Effect of genotype on outcome variable was compared to reference (wild type)

**Table 2:** *Plasmodium falciparum* (PCR) infection according to miRNA-146 genotype in placental blood of delivering primiparae

SNP rs2910164	Positive cases	Univariate analysis^a^		Multivariate analysis^b^	
	% (Fraction)	OR (95% CI)	*P*	aOR (95% CI)	***P***
Primiparae	64.9 (192/296)				
Wild type	60.4 (55/91)	1		1	
Heterozygote	60.8 (87/143)	1.0 (0.6–1.8)	1.0	1.4 (0.7–2.9)	0.38
Homozygote	80.6 (50/62)	2.7 (1.2–6.4)	0.013	5.9 (2.1–18.0)	0.0011
Het. or Hom.	66.8 (137/205)	1.3 (0.8–2.3)	0.29	2.1 (1.0–4.2)	0.038

Allele frequencies of SNP rs2910164 G > C were 0.49 (187/384) in infected (PCR) and 0.38 (80/208) in non-infected women (*P* = 0.02)

*OR* odds ratio, *aOR* adjusted odds ratio

^a^Fisher’s exact test for independence, compared to reference (wild type)

^b^Logistic regression model, including co-predictors age, season, PYR in urine or plasma and number of antenatal care visits. Effect of genotype on outcome variable was compared to reference (wild type)

**Table 3:** Past or present placental malaria (PCR, microscopy, haemozoin) according to miRNA-146 genotype in delivering primiparae

SNP rs2910164	Positive cases	Univariate analysis^a^		Multivariate analysis^b^	
	% (Fraction)	OR (95% CI)	P	aOR (95% CI)	P
Primiparae	67.9 (201/296)				
Wild type	61.5 (56/91)	1		1	
Heterozygote	66.4 (95/143)	1.2 (0.7–2.2)	0.48	1.6 (0.8–3.5)	0.21
Homozygote	80.6 (50/62)	2.6 (1.2–6.1)	0.013	5.9 (2.1–19.0)	0.0013
Het. or Hom.	72.2 (145/205)	1.5 (0.9–2.6)	0.14	2.3 (1.1–4.7)	0.020

*OR* odds ratio, *aOR* adjusted odds ratio

^a^Fisher’s exact test for independence, compared to reference (wild type)

^b^Logistic regression model, including co-predictors age, season, PYR in urine or plasma and number of antenatal care visits. Effect of genotype on outcome variable was compared to reference (wild type)

In peripheral blood samples of ANC attendees (mean age, 26.6 ± 6.3), malaria parasites were detected by microscopy in 32.8% (167/509), and 63.3% (322/509) were found to harbour *P. falciparum* by PCR. Infection prevalence (PCR) was higher in primigravidae (74.0%, 94/127) than in multigravidae (59.7%, 228/382; *P* = 0.004). The geometric mean parasite density (GMPD) of microscopically positive samples was 304/μL (95% CI, 259–356) for all ANC attendees, 165/μL (95% CI, 139–195) for primigravidae and 719/μL (95% CI, 536–963) for multigravidae. 14.7% (75/509) of the ANC attendees were febrile, and 53.6% (273/509) had anaemia (mean Hb, 10.7 ± 1.4 g/dL).

Among delivering primiparae, malaria parasites were detected microscopically in 26.4% (78/296) and 45.6% (135/296) of peripheral and placental blood films, respectively. By *P. falciparum* PCR, these figures were 59.1% (175/296) and 64.9% (192/296). Past or present placental malaria, i.e. considering also deposited hemozoin, was identified in 67.9% (201/296). The GMPD of microscopically positive peripheral and placental blood samples was 709/μL (95% CI, 563–894) and 1.15/HPF (95% CI, 0.86–1.54), respectively. 4.1% (12/293) of the delivering women were febrile and 38.8% (115/296) had anaemia (mean Hb, 11.2 ± 4.1 g/dL). 25.7% (76/296) of the neonates had LBW and 26.4% (78/296) were preterm.

Carriage of the miRNA-146a SNP was associated with increased odds of *P. falciparum* infection in both ANC attendees (Table 1) and primiparae (Tables 2, 3). This finding was due to a strong respective effect among homozygous individuals, i.e., more than five-fold increased odds of infection in primigravidae and primiparae, and a lesser, non-significant one among heterozygous women. Consequently, in primigravidae and primiparae, significant trends were seen for increasing *P. falciparum* prevalence from wild type individuals over heterozygous to homozygous women (ANC attendees, Z-statistic = − 2.8, *P *= 0.005; primigravidae, Z-statistic = − 2.5, *P *= 0.01; primiparae, Z-statistic = − 2.1, *P *= 0.04). Of note, increased *P. falciparum* prevalence was also observed among multigravid miRNA-146a SNP carriers, however, only weakly and statistically not significant.

In terms of clinical manifestation of infection, the miRNA-146a SNP did not show any significant association. In ANC attendees, fever occurred in 17.1% (28/164) of wildtype individuals and 13.6% (47/345; *P *= 0.3) of SNP carriers, and anaemia was present in 49.4% (81/164) and 55.7% (192/345; *P* = 0.2), respectively. Likewise, among primiparae, proportions did not differ between wildtype women and SNP carriers for fever (5.6%, 5/89 vs. 3.4%, 7/204; *P *= 0.5), anaemia (40.7%, 37/91 vs. 38.0, 78/205; *P *= 0.7; LBW (25.3%, 23/91 vs. 25.8%, 53/205; *P *= 1.0), and preterm delivery (26.4%, 24/91 vs. 26.3%, 54/205; *P *= 1.0). Stratification by infection status did not change this observation.

## Discussion

A common miRNA-146a SNP is associated with increased odds of *P. falciparum* infection in first-time pregnant women. This suggests this regulator of inflammation and innate immune responses to be involved in susceptibility to malaria. Genetic host variation contributes to large inter-individual variation in susceptibility to and manifestation of malaria, and the high frequency of several alleles in malaria-endemic regions are considered to reflect evolutionary selection due to this disease. Examples of malaria-protective traits include haemoglobin variants, enzyme disorders, and erythrocyte membrane polymorphisms [20]; whereas polymorphisms in genes encoding innate immune factors may increase or decrease susceptibility and manifestation [21].

The present study for the first time shows an impact of a miRNA genetic variation on the risk of human malaria, even though functional investigations have previously pointed to a role of miRNAs in that disease [22–24]. As a limitation, the present cross-sectional studies were not a priori designed to show associations with genetic traits. As a matter of fact, association does not necessarily mean causality. The classification of past or present placental malaria, i.e., combining microscopy, hemozoin detection, and PCR results, was applied to yield the highest diagnostic sensitivity including recently resolved infection (hemozoin) but does not match with the otherwise known classification based on placental histopathology. Lastly, due to the absence or late development of acquired immune mechanisms targeting the specific malaria parasites adhering to the intervillous syncytiotrophoblast [2], primigravidae and primiparae are considered relatively immune-naive. On the one hand, this facilitates the identification of the influence of genetic disposition, particularly with respect to innate immune responses. Therefore, and after having observed only weak and nonsignificant associations among multigravid ANC attendees, we abstained from genotyping multiparae. On the other hand, these findings need to be confirmed for other diseases entities, e.g. uncomplicated malaria or severe paediatric malaria.

Both TLR-2 and TLR-4 recognize *P. falciparum*, which initiates innate immune responses [25]. During innate recognition, miRNA-146a is up-regulated by NF-kB through a MyD88-dependent pathway. Subsequently, IRAK-1 and TRAF-6 are downregulated by miRNA-146a through posttranslational repression. MiRNA-146a thus influences TLR functionality via a negative feedback loop on the downstream mediators IRAK-1 and TRAF-6 [6, 7].

Consequently, altered TLR and cytokine signalling might influence the innate immune response to *P. falciparum* in individuals with variant miRNA-146a. The miRNA-146a rs2910164 G > C SNP, located in the passenger strand of the hairpin structured miRNA (miRNA-146a*), affects the processing of pre-miRNA-146a into mature miRNA-146a. Homozygosity for this polymorphism is associated with reduced expression of the downstream mediators, and heterozygosity with the expression of additional miRNA-146a: one from the leading strand and two from the passenger strand (miRNA-146a*G and miRNA-146a*C), which all three give rise to a mature miRNA [26, 27]. The additional mature miRNA-146a*G and miRNA-146a*C are predicted to have a distinct set of target genes, different from the mature miRNA-146a [26]. Whereas no results with respect to malaria have been published, previous studies reported associations of miRNA-146a rs2910164 G > C with increased susceptibility to pulmonary tuberculosis [12] and leprosy [13], in addition to various effects in neoplastic conditions [10].

Expanding on Haldane’s malaria hypothesis, a polymorphism increasing malaria risk should be expected to be rare in endemic regions. However, in sub-Saharan Africa, miRNA-146a rs2910164 occurs in 67% (GC, 44.2%; CC, 23.0%) [28], similar to the present results, and thus more frequently than in Caucasians (41%; GC, 34.5%; CC, 6.2%) [28]. Similar discrepancies have been observed for, e.g., *TLR*-*4* variants or mannose-binding lectin deficiency [29, 30].

Potential explanations include alleles or genotypes, which may have become deleterious after the out-of-Africa-migration of humans, possibly because of increased susceptibility to severe bacterial infections and sepsis [31]. Alternatively, counter-selecting evolutionary forces leading to high miRNA-146a SNP frequencies in sub-Saharan Africa (which consequently would have a larger impact than malaria) are hard to imagine. With respect to tuberculosis, both increased and decreased susceptibility to pulmonary tuberculosis in case of miRNA-146a rs2910164 have been reported from China [12, 32]. Moreover, the present study showed associations with infection but not with manifestation. For a common *TLR*-*4* SNP in Ghana, increased susceptibility to severe malaria but a trend towards reduced mortality was found in a previous study [30]. Considering the complex roles of miRNA-146a in immunomodulation and inflammatory responses [33], more refined and prospective studies involving patients of differing ethnicities are required to disentangle the potential influences of miRNA-146a rs2910164 G > C on the various entities of malaria, i.e., from (asymptomatic) infection to (fatal) disease.

## Conclusion

Homozygosity for the miRNA-146a rs2910164 SNP predisposes to *P. falciparum* infection in first-time pregnant Ghanaian women. This suggests that miRNA-146a plays an important role in the respective innate immune response but further studies are required to detail the actual pathophysiology involved. Understanding protective immunity towards malaria in pregnancy is essential to improve maternal health and for decreasing the huge share of malaria in infant mortality in sub-Saharan Africa. MiRNA-based biopharmaceuticals are an active field of research. Enhanced antimicrobial immune responses have been observed after silencing or administration of miRNAs [8, 9, 34, 35]. The findings in the present study suggest that miRNA-146a is involved in innate immunity against malaria highlighting its potency as a biopharmaceutical target.

## Abbreviations

ANC: antenatal care
aOR: adjusted odds ratio
CI: confidence interval
GMPD: geometric mean parasite density
Hb: haemoglobin
HPF: high power fields
IRAK-1: interleukin-1 receptor-associated kinase
LBW: low birth weight
miRNA: micro-RNA
OR: odds ratio
PYR: pyrimethamine
SNP: single nucleotide polymorphism
TLR: toll-like receptors
TNF: tumor necrosis factor
TRAF-6: TNF receptor-associated factor-6
WBC: white blood cell

## References

[1] Rogerson SJ, Desai M, Mayor A, Sicuri E, Taylor SM, van Eijk AM (2018). Burden, pathology, and costs of malaria in pregnancy: new developments for an old problem. Lancet Infect Dis..

[2] Ricke CH, Staalsoe T, Koram K, Akanmori BD, Riley EM, Theander TG (2000). Plasma antibodies from malaria-exposed pregnant women recognize variant surface antigens on *Plasmodium falciparum*-infected erythrocytes in a parity-dependent manner and block parasite adhesion to chondroitin sulfate A. J Immunol..

[3] Mockenhaupt FP, Ulmen U, von Gaertner C, Bedu-Addo G, Bienzle U (2002). Diagnosis of placental malaria. J Clin Microbiol.

[4] Mockenhaupt FP, Hamann L, von Gaertner C, Bedu-Addo G, von Kleinsorgen C, Schumann RR (2006). Common polymorphisms of toll-like receptors 4 and 9 are associated with the clinical manifestation of malaria during pregnancy. J Infect Dis.

[5] Esposito S, Molteni CG, Zampiero A, Baggi E, Lavizzari A, Semino M (2012). Role of polymorphisms of toll-like receptor (TLR) 4, TLR9, toll-interleukin 1 receptor domain containing adaptor protein (TIRAP) and FCGR2A genes in malaria susceptibility and severity in Burundian children. Malar J..

[6] Mehta A, Baltimore D (2016). MicroRNAs as regulatory elements in immune system logic. Nat Rev Immunol.

[7] Taganov KD, Boldin MP, Chang KJ, Baltimore D (2006). NF-kappaB-dependent induction of microRNA miR-146, an inhibitor targeted to signaling proteins of innate immune responses. Proc Natl Acad Sci USA.

[8] Alexander M, Hu R, Runtsch MC, Kagele DA, Mosbruger TL, Tolmachova T (2015). Exosome-delivered microRNAs modulate the inflammatory response to endotoxin. Nat Commun..

[9] Ho BC, Yu IS, Lu LF, Rudensky A, Chen HY, Tsai CW (2014). Inhibition of miR-146a prevents enterovirus-induced death by restoring the production of type I interferon. Nat Commun..

[10] Hao X, Xia L, Qu R, Yang X, Jiang M, Zhou B (2018). Association between miR-146a rs2910164 polymorphism and specific cancer susceptibility: an updated meta-analysis. Fam Cancer.

[11] Park R, Lee WJ, Ji JD (2016). Association between the three functional miR-146a single-nucleotide polymorphisms, rs2910164, rs57095329, and rs2431697, and autoimmune disease susceptibility: a meta-analysis. Autoimmunity..

[12] Li D, Wang T, Song X, Qucuo M, Yang B, Zhang J (2011). Genetic study of two single nucleotide polymorphisms within corresponding microRNAs and susceptibility to tuberculosis in a Chinese Tibetan and Han population. Hum Immunol.

[13] Cezar-de-Mello PF, Toledo-Pinto TG, Marques CS, Arnez LE, Cardoso CC, Guerreiro LT (2014). Pre-miR-146a (rs2910164 G > C) single nucleotide polymorphism is genetically and functionally associated with leprosy. PLoS Negl Trop Dis..

[14] Browne EN, Frimpong E, Sievertsen J, Hagen J, Hamelmann C, Dietz K (2000). Malariometric update for the rainforest and savanna of Ashanti region, Ghana. Ann Trop Med Parasitol..

[15] Mockenhaupt FP, Bedu-Addo G, von Gaertner C, Boye R, Fricke K, Hannibal I (2006). Detection and clinical manifestation of placental malaria in southern Ghana. Malar J..

[16] Mockenhaupt FP, Rong B, Till H, Eggelte TA, Beck S, Gyasi-Sarpong C (2000). Submicroscopic *Plasmodium falciparum* infections in pregnancy in Ghana. Trop Med Int Health..

[17] Snounou G, Viriyakosol S, Zhu XP, Jarra W, Pinheiro L, do Rosario VE (1993). High sensitivity of detection of human malaria parasites by the use of nested polymerase chain reaction. Mol Biochem Parasitol..

[18] WHO (1972). Nutritional anemias. Report of a WHO group of experts. World Health Organ Tech Rep Ser.

[19] Finnstrom O (1977). Studies on maturity in newborn infants. IX Further observations on the use of external characteristics in estimating gestational age. Acta Paediatr Scand.

[20] Kwiatkowski DP (2005). How malaria has affected the human genome and what human genetics can teach us about malaria. Am J Hum Genet.

[21] de Mendonça VR, Goncalves MS, Barral-Netto M (2012). The host genetic diversity in malaria infection. J Trop Med..

[22] Chamnanchanunt S, Kuroki C, Desakorn V, Enomoto M, Thanachartwet V, Sahassananda D (2015). Downregulation of plasma miR-451 and miR-16 in *Plasmodium vivax* infection. Exp Parasitol.

[23] LaMonte G, Philip N, Reardon J, Lacsina JR, Majoros W, Chapman L (2012). Translocation of sickle cell erythrocyte microRNAs into *Plasmodium falciparum* inhibits parasite translation and contributes to malaria resistance. Cell Host Microbe.

[24] Chamnanchanunt SOK, Desakorn V, Koga M, Nakamura Y, Shiotsu H (2015). Down-regulated micrornas in plasma and red blood cells of patients with malaria infection. Exp Hematol.

[25] Krishnegowda G, Hajjar AM, Zhu J, Douglass EJ, Uematsu S, Akira S (2005). Induction of proinflammatory responses in macrophages by the glycosylphosphatidylinositols of *Plasmodium falciparum*: cell signaling receptors, glycosylphosphatidylinositol (GPI) structural requirement, and regulation of GPI activity. J Biol Chem.

[26] Jazdzewski K, Liyanarachchi S, Swierniak M, Pachucki J, Ringel MD, Jarzab B (2009). Polymorphic mature microRNAs from passenger strand of pre-miR-146a contribute to thyroid cancer. Proc Natl Acad Sci USA.

[27] Jazdzewski K, Murray EL, Franssila K, Jarzab B, Schoenberg DR, de la Chapelle A (2008). Common SNP in pre-miR-146a decreases mature miR expression and predisposes to papillary thyroid carcinoma. Proc Natl Acad Sci USA.

[28] dbSNP summary of Genotypes for ss52076591.https://www.ncbi.nlm.nih.gov/projects/SNP/snp_ss.cgi?ss=ss52076591. Accessed 28 Nov 2018.

[29] Holmberg V, Schuster F, Dietz E, Sagarriga Visconti JC, Anemana SD, Bienzle U (2008). Mannose-binding lectin variant associated with severe malaria in young African children. Microbes Infect.

[30] Mockenhaupt FP, Cramer JP, Hamann L, Stegemann MS, Eckert J, Oh NR (2006). Toll-like receptor (TLR) polymorphisms in African children: common TLR-4 variants predispose to severe malaria. Proc Natl Acad Sci USA.

[31] Ferwerda B, McCall MB, Alonso S, Giamarellos-Bourboulis EJ, Mouktaroudi M, Izagirre N (2007). TLR4 polymorphisms, infectious diseases, and evolutionary pressure during migration of modern humans. Proc Natl Acad Sci USA.

[32] Zhang X, Li Y, Li X, Zhang W, Pan Z, Wu F (2015). Association of the miR-146a, miR-149, miR-196a2 and miR-499 polymorphisms with susceptibility to pulmonary tuberculosis in the Chinese Uygur, Kazak and Southern Han populations. BMC Infect Dis.

[33] Saba R, Sorensen DL, Booth SA (2014). MicroRNA-146a: a dominant, negative regulator of the innate immune response. Front Immunol..

[34] Wang X, Gu H, Qin D, Yang L, Huang W, Essandoh K (2015). Exosomal miR-223 contributes to mesenchymal stem cell-elicited cardioprotection in polymicrobial sepsis. Sci Rep..

[35] Tay HL, Kaiko GE, Plank M, Li J, Maltby S, Essilfie AT (2015). Antagonism of miR-328 increases the antimicrobial function of macrophages and neutrophils and rapid clearance of non-typeable *Haemophilus influenzae* (NTHi) from infected lung. PLoS Pathog.

